# Side Lighting Enhances Morphophysiology by Inducing More Branching and Flowering in Chrysanthemum Grown in Controlled Environment

**DOI:** 10.3390/ijms222112019

**Published:** 2021-11-06

**Authors:** Jingli Yang, Byoung Ryong Jeong

**Affiliations:** 1 Department of Horticulture, Division of Applied Life Science (BK21 Four), Graduate School of Gyeongsang National University, Jinju 52828, Korea; yangmiaomiaode@gmail.com; 2 Institute of Agriculture and Life Science, Gyeongsang National University, Jinju 52828, Korea; 3 Research Institute of Life Science, Gyeongsang National University, Jinju 52828, Korea

**Keywords:** chrysanthemum, lighting direction, branching, flowering, photomorphogenesis, photosynthesis, phototropism, stomatal properties

## Abstract

Light is one of the most important factors that influence plant growth and development. This study was conducted to examine how lighting direction affects plant morphophysiology by investigating plant growth parameters, leaf anatomy, epidermal cell elongation, stomatal properties, chloroplast arrangement, and physiological changes. In closed-type plant factory units, the rooted cuttings of two chrysanthemum (*Chrysanthemum morifolium* Ramat.) cultivars, ‘Gaya Glory’ and ‘Pearl Egg’, were subjected to a 10 h photoperiod with a 300 μmol∙m^−2^∙s^−1^ photosynthetic photon flux density (PPFD) provided by light-emitting diodes (LEDs) from three directions relative to the plant including the top, side, and bottom. Compared to the top or bottom lighting, the side lighting greatly enhanced the plant growth, improved the leaf internal structure and chloroplast arrangement, induced small stomata with a higher density, and promoted stomatal opening, which is associated with an increased stomatal conductance and photosynthetic efficiency. It is worth noting that the side lighting significantly enhanced the induction of branching and flowering for both cultivars., The plants grown with side lighting consistently exhibited the greatest physiological performance. We conclude that the lighting direction had a profound effect on the morphophysiological characteristics of chrysanthemum, and that side lighting dramatically promoted their growth and development, especially in their branching and flowering.

## 1. Introduction

Growing plants indoors through means such as the plant growth chamber, plant factory, and greenhouse using supplemental lighting provided by highly controllable artificial lighting systems, can circumvent the influence of unstable external environmental factors, and has become a popular methodology used in horticulture research. Such research primarily aims improve the quality and quantity of agricultural products. The applications of Plant Artificial Lights (PALs) means that sunlight does not constitute the unique light source for agricultural production as it can be replaced by PALs. Indoor plant cultivation with artificial lighting is an innovative technology for modern agriculture that fundamentally changes the concept of farming [1]. However, growing plants indoors presents challenges that all growers must overcome. For these reasons, the methods through which to select the correct lighting for indoor plant cultivation has become a subject of great interest.

Firstly, lighting is the crucial factor for plant growth. Plants cannot migrate away from causes of stress or seek out a site with the ideal environmental conditions, since they are sessile and photoautotrophic [2]. Instead, they must alter their growth processes to survive and reproduce in their environment. Many species have evolved complex photosensory systems that allow them to react appropriately to their surroundings [3]. Photosynthesis and many other physiological processes connected to plant development require light as the primary source of energy, and plants are very sensitive to this essential external signal [4]. Plant survival is largely dependent on light adaptation [5]. More sophisticated plants have evolved a specialized skill known as photomorphogenesis, which allows them to alter their shapes in response to changing light circumstances [6].

Moreover, the position of the indoor growth lights is crucial to the overall success of plants. The following two factors explain the importance of the lighting position: (1) the number of plants that the light effectively covers, and plant shade avoidance. A light’s footprint refers to the area that it illuminates. There is an effective coverage rate for each indoor growth light. However, not all light within the light’s footprint is of equal intensity, because the edges will receive less intense light, and the center will receive more direct light. If a large number of plants are placed under a single light, the outlier plants yield significantly less light than those directly underneath the growth light. This directly impacts the number of plants that can be grown efficiently. Furthermore, shade-avoidance responses are changes in the plant body structure and functions that occur in response to light cues supplied by nearby plants, which attempt to reduce present or future shade [7]: by increasing their leaf area and the amount of the chlorophyll a/b light-harvesting complex, so as to improve their light-harvesting capabilities [8], or by adapting their morphology to position their leaves outside of the shade. Plants usually respond to shade by elongating their stem-like organs, such as hypocotyls and leaf petioles, as well as orienting their leaves in a more upward manner [9,10]. Additionally, reduced branching also occurred in plants that were grown under shade conditions [11]. (2) The intensity of light that is received is also important. For crop physiology and biochemistry, the light intensity is one of the most important environmental variables [12]. Even a small increase or reduction in light intensity causes significant changes in the leaf form and structure in most agricultural plants [13]. In addition, in low light circumstances, the dry matter of the roots, stems, leaves, and of the whole plant decreased, as does the photosynthetic rate, transpiration, stomatal conductance, and stem diameter [14,15]. Overall, the light intensity is one of the most important variables in controlling the plant processes including germination, leaf proliferation and expansion, photosynthesis, bud and flower initiation, and cell division [16,17,18].

Variations in the light positioning affect the plant morphophysiology. The leaf orientation directly determines the light interception. It has been postulated that changes in the leaf angle and leaf movement (epinastic or hyponastic), induced by phototropism, could help to provide a higher photosynthetic capacity and efficiency in plants [19]. Usually, low light or shade conditions induce the leaf movement responses in plants [20,21,22], which are controlled by the phytochrome and cryptochrome pathways [23,24,25,26]. Therefore, changes in the leaf angle are believed to be a typical morphological response of the shade-avoidance syndrome, which allows plants to capture more sunlight and increases the carbon gain in light competition conditions [27,28,29]. Due to changes in the leaf angle, the epidermal cells in midribs are stimulated, which further affects the stomatal state. The stomatal density and size are regarded as indicators of a plants’ acclimation and adaptation to contrasting environments [30,31]. Photosynthesis is necessary for stomatal opening, and the guard cells of etiolated leaves do not have chlorophyll and cannot carry out photosynthesis, so stomatal movement does not occur under the influence of light [32,33]. Moreover, because increased stomatal density decreases the risk of stomatal injury from various stresses, having a highly dense and open small stoma is the best strategy for obtaining the highest stomatal conductance at low CO_2_ concentrations, and is valuable for a high photosynthetic efficiency [34]. Consistent with our past studies, the lighting direction affected the plant morphology, as evidenced by the growth parameters, epidermal cell elongation, stomatal properties, and physiological changes with the changing light directions [35]. Thus, these findings indicate a strong relationship between the leaf angle and lighting conditions. Moreover, it is important to investigate the responses of the plant leaf angle under changing light directions to understand how the growth and development parameters of chrysanthemums are affected by different lighting directions.

Plants have developed sophisticated acclimatization strategies to overcome unfavorable conditions, such as the ROS (reactive oxygen species) scavenged enzymatic antioxidant system [36]. Under this condition, the balance between the production of ROS and the quenching activity of antioxidants becomes disrupted, often resulting in oxidative damage [37]. Usually, a greater ability to eliminate ROS indicates higher antioxidant enzyme activity. Moreover, chlorophyll a is more sensitive to ROS than chlorophyll b under stress conditions. ROS directly causes the degradation of chlorophyll a, and the total chlorophyll contents [38,39], which affects photosynthesis. Moreover, the metabolic capacity of photosynthesis in plants has been evaluated through the quantity and activity of key enzymes involved in the CO_2_ fixation and the regeneration of RuBisCO-1, 5-bisphosphate (RuBP) under various circumstances [40,41,42], as well as the content and activity of light-capturing components, electron transport fragments, and energy transferring enzymes [43,44,45]. RuBisCO (RuBP carboxylase or oxygenase) catalyzes the CO_2_ fixation in photosynthesis [46], which is directly engaged in the first phase of the Calvin Benson cycle and accounts for 12–35% of the leaf proteins, most notably in C_3_ crop plants [47]. The primary biochemical constraint implicated in the shade-associated down-regulation of the net photosynthetic rate has been identified in previous reports as a decrease in the RuBisCO quantity or activity [40].

Chlorophyll fluorescence measurements have been acknowledged as a valuable and informative indicator for evaluating various photosynthetic light responses. The investigation and determination of the key traits of this technique has attracted much attention [48]. Chlorophyll fluorescence is primarily and successfully utilized to determine the possible quantum yield of photosystem II and photoinhibition under prevailing light and shade environments [49]. Shade has a substantial impact on the performance and structure of the photosynthetic apparatus [50]. It reduces the thickness of leaves, palisades, and spongy tissues, and inhibits energy transfer from PSII to PSI, resulting in a reduced chlorophyll fluorescence [13,50].

Numerous studies have confirmed that the light intensity, quality, duration, photoperiod, and sources such as the sun or artificial light sources, i.e., light-emitting diodes (LED), metal halide (MH), or high-pressure sodium (HPS) light, and photoperiod significantly influence plant growth and development [12,51,52]. However, researchers have rarely paid attention to the impact of different light directions on plant morphophysiology, especially of the bottom lighting. In previous studies with lettuce species, plants adapted to the different lighting directions from the cellular level to the whole plant level, particularly in leaf morphology and root production [35]. Nevertheless, there is no obvious stem in lettuces. In this study, two species of chrysanthemum were considered, which is an obligate short-day flowering plant with a distinct stem. We investigated how a chrysanthemum responds to the different lighting directions to help fine-tune the growth environment for their development. Furthermore, our study refers to the profound effects on the positioning and internal structure of leaves, the changes in the chloroplast arrangement, photosynthetic and chlorophyll fluorescence parameters, leaf carbon status through sucrose and starch contents, the activity of key enzymes related to photosynthesis and sugar synthesis, and a transcriptional analysis of some targeted genes to investigate the optimum lighting direction for the growth and development of chrysanthemum plants. Most interestingly, branching and flowering were visibly induced by side lighting. We believe these findings can be verified by studies on plant morphophysiology, aesthetics, and form, as well as the commercial value of ornamental plants considering the space and lighting efficiency in plant production in closed environments using artificial light, such as plant growth chambers and plant factories. Finally, we concluded that changes in the morphophysiology of chrysanthemum plants are tightly related to the changes in the lighting direction.

## 2. Results

### 2.1. Morphological Characteristics and Growth Parameters

**Figure 1:**
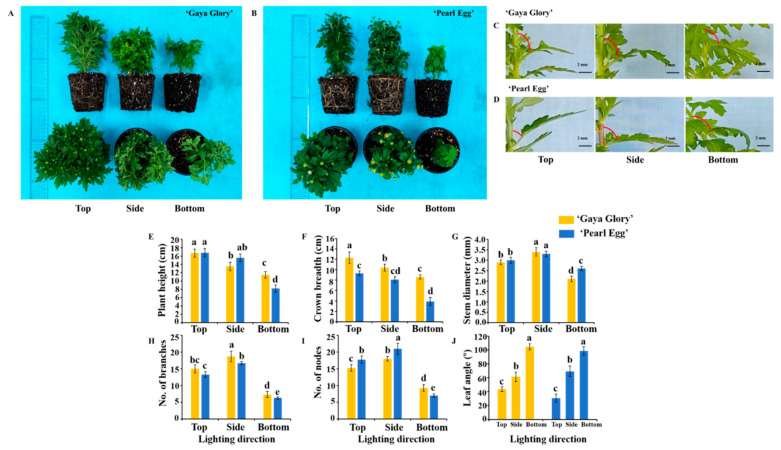
Changes in the phenotype and plant traits of chrysanthemum ‘Gaya Glory’ and ‘Pearl Egg’ plants as affected by the lighting direction after 45 days of cultivation. The plant morphology (**A**,**B**), leaf angle (**C**,**D**), plant height (**E**), crown breadth (**F**), stem diameter (**G**), No. of branches (**H**) and nodes (**I**), and adaxial leaf petiole angle (**J**) of chrysanthemum ‘Gaya Glory’ and ‘Pearl Egg’ plants grown with different lighting directions for 45 days, respectively. Vertical bars indicate the means ± standard error (*n* = 3). Different lowercase letters indicate significant separations within treatments by Duncan’s multiple range test at *p* ≤ 0.05. Bars indicate 2 mm.

Figure 1 presents the morphological and growth characteristics of the chrysanthemum plants with different lighting directions. In the present experiment, the phenotype and growth attributes of chrysanthemum plants ‘Gaya Glory’ and ‘Pearl Egg’, including the plant height, crown breadth, stem diameter, number of branches and nodes, and adaxial leaf petiole angle, were significantly affected by the lighting direction. The plant height and crown breadth of chrysanthemum plants considerably increased with the top lighting compared to the bottom lighting, regardless of the cultivar. Specifically, the maximum plant heights of 16.7 and 16.8 cm and crown breadths of 12.3 and 9.7 cm resulted from lighting from the top, and the minimum plant heights of 12.5 and 8.2 cm and crown breadths of 8.6 and 3.9 cm were observed with the bottom lighting in chrysanthemum ‘Gaya Glory’ and ‘Pearl Egg’, respectively. Relative to the bottom lighting, stem diameter as well as the number of branches and nodes of ‘Gaya Glory’ were improved by 61.9, 156.2, and 93.6% with the side lighting. Similarly, the same attributes in the ‘Pearl Egg’ experienced an increase of 19.2, 165.1, and 192.3%, respectively with the side lighting. Relatively smaller changes were observed with the top lighting, with improvements of only 17.2, 24.7, and 17.7% in ‘Gaya glory’ and only 6.7, 25.6, and 20.5% in ‘Pearl Egg’. The leaf angle of chrysanthemum plants was considerably higher when light was supplied from the bottom. The maximum leaf angles of 104.6 and 98.7° were measured with the bottom lighting, while the minimum leaf angles of 43.81 and 30.76° were observed with the top lighting in ‘Gaya Glory’ and ‘Pearl Egg’, respectively. Overall, lighting from the side or the top remarkably increased the plant growth and development, in comparison with lighting from the bottom. However, the bottom lighting markedly increased the leaf angle and further resulted in chrysanthemum leaves bending downward due to the phototaxis in both of the chrysanthemum cultivars. 

Table 1 presents further details on the growth and development parameters which were measured after 45 days of cultivation. For both ‘Gaya Glory’ and ‘Pearl Egg’, the observed differences in growth and development parameters under different lighting directions displayed a similar tendency. The top lighting significantly increased the length of the internode, and the greatest shoot fresh and dry weights were obtained with lighting from the side. The shoot fresh and dry weights were dramatically reduced when using the bottom lighting. Compared with the bottom lighting, the top and side lighting significantly enhanced the number of leaves, and the side lighting was resulted in a much more dramatic increase. The leaf width, using bottom lighting, was lower than with the top lighting, whereas the opposite trend was observed in the leaf length. There were no significant differences in the duration of treatment to the visible flower buds (DVB) between plants grown using lighting from the top or the side. Singularly, the bottom lighting resulted in no flowering in both ‘Gaya Glory’ and ‘Pearl Egg’. In addition, compared to lighting from the top, the side lighting dramatically enhanced the flower number. The length, fresh and dry weights of the roots exhibited highly positive influences of the side lighting application. Moreover, the shoot to root fresh weight ratio and dry weight ratio significantly increased with the top lighting, whereas the lowest ratios were observed for the lighting from the bottom. The *F*-test data simultaneously exhibited significant differences in response to the three lighting directions. It was also demonstrated that the extent of the effects of the different lighting directions on the growth parameters was strongly dependent on the cultivar, although the overall trends were similar.

### 2.2. Leaf Anatomy

**Figure 2:**
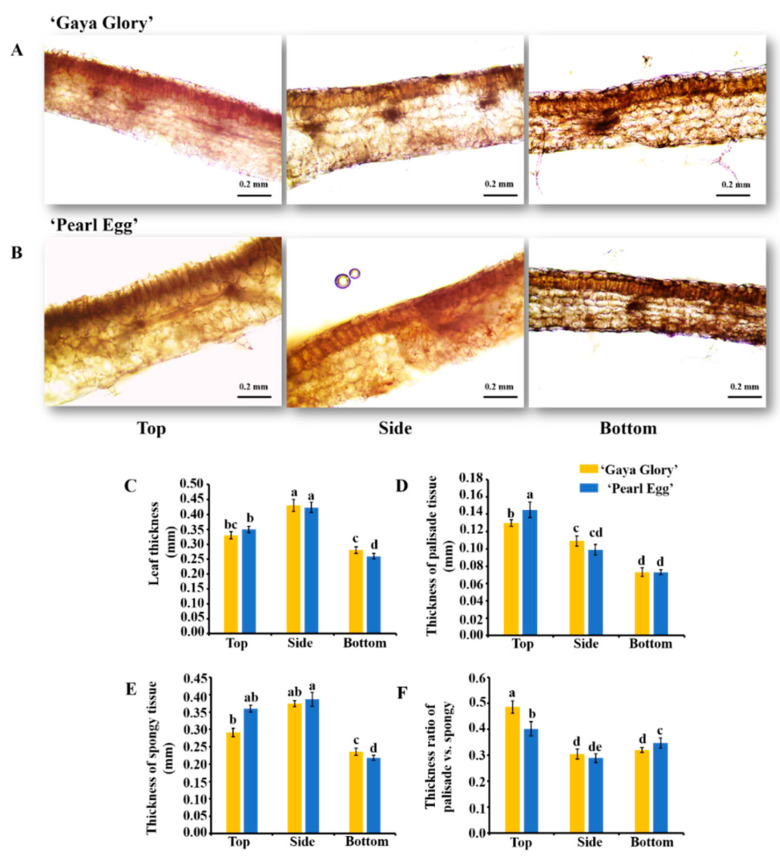
Changes in the leaf structure of chrysanthemum ‘Gaya Glory’ and ‘Pearl Egg’ plants as affected by the lighting direction after 45 days of cultivation. The picture representation of the leaf structure (**A**,**B**), leaf thickness (**C**), palisade thickness (**D**), spongy tissue thickness (**E**), and thickness ratio of palisades and spongy tissues (**F**) of chrysanthemum ‘Gaya Glory’ and ‘Pearl Egg’ plants under different lighting directions 45 days, respectively. Vertical bars indicate the means ± standard error (*n* = 3). Different lowercase letters indicate the significant separation within treatments by Duncan’s multiple range test at *p* ≤ 0.05. Bars indicate 0.2 mm.

To further explore the influence of lighting direction on the leaf structure, the leaf anatomy was investigated in chrysanthemum ‘Gaya Glory’ and ‘Pearl Egg’ plants. In this study, significant differences in the leaf thickness, palisade tissues thickness, spongy tissue thickness, and the ratio of palisade and spongy tissue thicknesses were examined in relation to the three lighting directions specified (Figure 2A–F). Interestingly, chrysanthemum leaves displayed perfectly developed palisade tissues with the top lighting, whereas clearer and more compact structures of spongy tissues were observed with the bottom lighting. Moreover, the leaf thickness and spongy tissue thickness of chrysanthemum leaves with the side lighting were both significantly higher than those subject to the bottom lighting. However, the chrysanthemum plants grown with the top lighting exhibited the greatest thickness of the palisade tissues. In particular, the increased ratio of palisade and spongy tissue thicknesses were observed with the top lighting, regardless of the cultivar. These findings indicated that non-optimal lighting directions negatively affects the chrysanthemum leaf tissue structures, while an optimum lighting direction positively influences the chrysanthemum leaf tissue structures.

### 2.3. Morphology of the Epidermal Cells and Stomata

**Figure 3:**
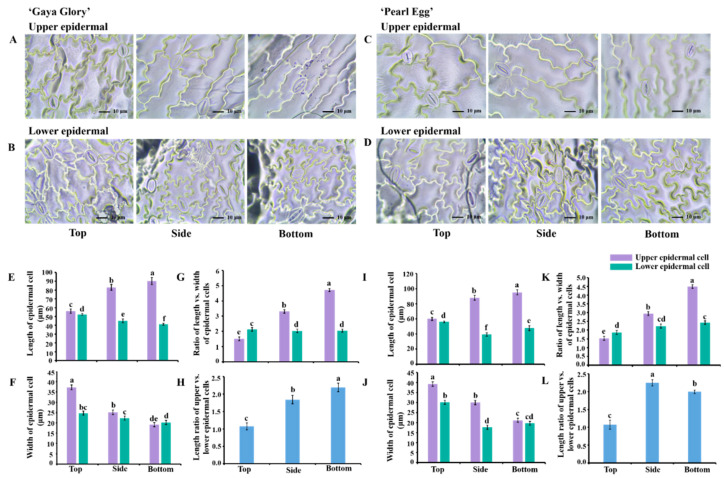
Changes in the upper and lower epidermal cell morphology of chrysanthemum ‘Gaya Glory’ and ‘Pearl Egg’ plants as affected by the lighting direction after 45 days of cultivation. Upper and lower epidermal cell morphology of ‘Gaya Glory’ (**A**,**B**) and ‘Pearl Egg’ (**C**,**D**), cell length and width, the ratio of cell length to width, and the ratio of the upper and lower epidermal cell lengths of ‘Gaya Glory’ (**E**–**H**) and ‘Pearl Egg’ (**I**–**L**). Vertical bars indicate the means ± standard error (*n* = 3). Different lowercase letters indicate significant separations within treatments by Duncan’s multiple range test at *p* ≤ 0.05. Bars indicate 10 μm.

The lighting direction displayed a significant influence on the morphology in the epidermal cells of the leaf midribs during their development (Figure 3A–D). In the ‘Gaya Glory’, the upper epidermal cell length and width were greatly promoted with lighting from the bottom and top, respectively. Moreover, the largest values of the ‘upper epidermal cell length/width’ and ‘upper/lower epidermal cell length’ were observed with the bottom lighting, and similar tendencies were observed in ‘Pearl Egg’ as those observed in ‘Gaya Glory’. In addition, there were relatively milder or no significant changes in the lower epidermal cells in response to the three lighting directions, regardless of the cultivar.

**Figure 4:**
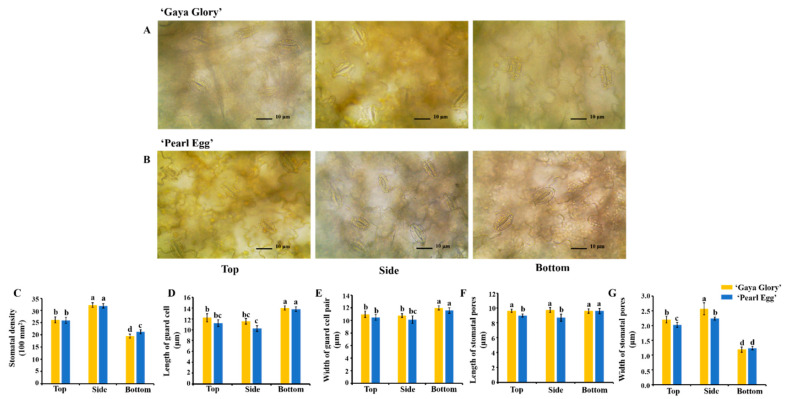
Changes in the stomatal micrographs of chrysanthemum ‘Gaya Glory’ (**A**) and ‘Pearl Egg’ (**B**) leaves, and stomatal density (**C**), guard cell length (**D**), width of guard cell pair (**D**), pore length (**F**) and width (**G**) of ‘Gaya Glory’ and ‘Pearl Egg’, respectively, as affected by the lighting direction after 45 days of cultivation. Vertical bars indicate the means ± standard error (*n* = 3). Different lowercase letters indicate significant separations within treatments by Duncan’s multiple range test at *p* ≤ 0.05. Bars indicate 10 μm.

To further explore the changes in the epidermal cellular morphology, we examined the stomatal state in chrysanthemum leaves as affected by the different lighting directions. The stomatal properties of chrysanthemum leaves were strongly affected by the lighting direction (Figure 4). Lighting from the side significantly increased the stomatal density and pore width in both of the chrysanthemum cultivars (Figure 4C,G), whereas the stomatal size was negatively affected by the side lighting (Figure 4A,B). In ‘Gaya Glory’, the bottom lighting markedly increased the stomatal size and inhibited stomatal opening, while there were no significant differences in the stomatal state in response to the top and side lighting. Similar trends were observed in ‘Pearl Egg’. The stomatal pore length was mildly increased when the bottom lighting was used, as compared to that with the top and side lighting in ‘Pearl Egg’ leaves, while that of ‘Gaya Glory’ was not significantly affected by the lighting direction (Figure 4D–F). Our results indicated that the side lighting resulted in a much more prominent increase in the stomatal density and stomatal opening, although it significantly decreased the stomatal size when compared to the bottom lighting. Overall, different lighting directions resulted in different changes in the chrysanthemum leaf phenotype and further affected the morphology of the epidermal cells and stomata, and eventually led to changes in the stomatal properties.

### 2.4. Chloroplast Distribution and Chlorophyll Content

**Figure 5:**
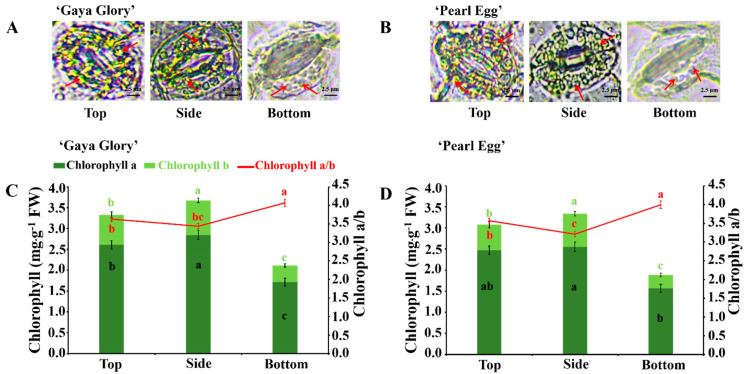
Changes in the chloroplast structure/redundancy (**A**,**B**) and chlorophyll content (Chl a and Chl b) and Chl a to b ratio (**C**,**D**) of chrysanthemum ‘Gaya Glory’ and ‘Pearl Egg’ leaves, respectively, as affected by the lighting direction after 45 days of cultivation. Vertical bars indicate the means ± standard error (*n* = 3). Different lowercase letters indicate significant separations within treatments by Duncan’s multiple range test at *p* ≤ 0.05. Bars indicate 2.5 μm.

The lighting direction exhibited significant influences on the stomatal properties. In this study, the chloroplasts, which are located in the guard cells of chrysanthemum leaves, were examined (Figure 5A,B). In both ‘Gaya Glory’ and ‘Pearl Egg’, a poor distribution of chloroplasts was observed with the bottom lighting, while an abundance of chloroplasts were distributed in both the top and side lighting treatments. Moreover, compared to that with the bottom lighting, the chloroplasts with the top and side lighting were clear and large, centrally organized in the cell, and showed a more compact arrangement. On the whole, the top and side lighting significantly improved the chloroplast structure and arrangement, regardless of the cultivar.

The lighting direction significantly affected the chlorophyll (Chl) content of the chrysanthemum leaves (Figure 5C,D). In this experiment, the lighting direction from the bottom uniformly and distinctly decreased the values of Chl a, Chl b, Chl a + b, while significantly increasing the Chl a/b in both cultivars. The greatest Chl a, Chl b, and Chl a + b contents, as well as the minimum Chl a/b, were observed with the side lighting. In addition, the Chl contents with the top lighting was mildly lower than with the side lighting but were always higher than with the bottom lighting. On average, the Chl a, Chl b, and Chl a + b contents increased by 66.3, 106.7, and 73.9% in ‘Gaya Glory’, and by 55.2, 110.3, and 68.2% in ‘Pearl Egg’ for the side lighting in comparison with the bottom lighting. These improvements suggest a direct relationship between the chlorophyll contents and the lighting direction.

### 2.5. Photosynthetic and Chlorophyll Fluorescence Characteristics

Table 2 shows the photosynthetic characteristics of chrysanthemum plants in response to the different light directions. The maximum *P*_n_, *T*_r_, *G*_s_, and *C*_i_ values of chrysanthemum plants were observed with either the top or side lighting. The *P*_n_, *T*_r_, *G*_s_, and *C*_i_ of chrysanthemum plants increased by 35.59, 46.46, 111.43, and 21.79% in ‘Gaya Glory’; and by 27.28, 68.47, 106.25, and 33.45% in ‘Pearl Egg’ with the top and side lighting, respectively, as compared to those of the bottom lighting. The increases in the net photosynthetic rate induced by the top and side lighting indicated that light direction is highly related to the photosynthesis. This relationship may be due to the enhancement of the stomatal properties, chloroplast redundancy, and chlorophyll content when top and side lighting were employed.

The absorbed radiation energy in chrysanthemum leaves was studied in response to the different lighting directions (Table 3). In this experiment, the chlorophyll fluorescence parameters including *F*_v_/*F*_m_, *F*_v_’/*F*_m_’, NPQ, and *qP* were significantly altered in response to the different lighting directions. Independent of the cultivar, the *F*_v_/*F*_m_, *F*_v_’/*F*_m_’, NPQ, and *qP* of chrysanthemum leaves with the top and side lighting were considerably higher than those for the bottom lighting, while insignificant differences were observed in response to the top and side lighting. Furthermore, the side lighting considerably increased the quantum yields of *F*_v_/*F*_m_, *F*_v_’/*F*_m_’, NPQ, and *qP* by 23.8, 38.5, 21.1, and 34.1%, respectively, in ‘Gaya Glory’; and by 22.7, 58.1, 46.1, and 58.3%, respectively in ‘Pearl Egg’, as compared to those with the bottom lighting. Overall, our results indicate that the provision of an appropriate lighting direction plays a crucial role in improving the chlorophyll fluorescence parameters and photosynthetic capacity of chrysanthemum plants.

### 2.6. Carbohydrates and Soluble Proteins

**Figure 6:**
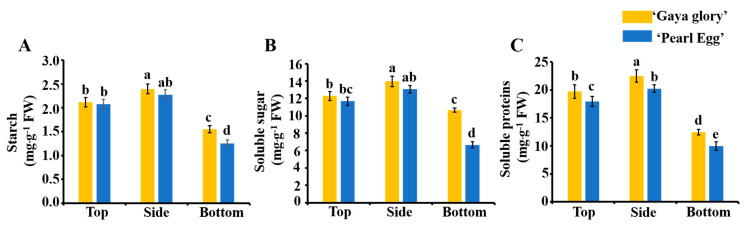
Changes in the content of carbohydrates (**A**,**B**) and soluble proteins (**C**) of chrysanthemum ‘Gaya Glory’ and ‘Pearl Egg’ leaves as affected by the lighting direction after 45 days of cultivation. Vertical bars indicate the means ± standard error (*n* = 3). Different lowercase letters indicate the significant separation within treatments by Duncan’s multiple range test at *p* ≤ 0.05.

The different lighting directions led to differences in photosynthetic efficiency (Table 2 and Table 3). To further investigate the effects of the lighting direction on photosynthesis in chrysanthemum, we determined the starch and total soluble sugar contents in the chrysanthemum leaves (Figure 6A,B). For ‘Gaya Glory’, as expected, the starch and total soluble sugar contents significantly increased when using the side lighting. The highest starch content of 2.4 mg g^−1^ and total soluble sugar content of 14.0 mg g^−1^ were measured with the side lighting. The same trend was observed in ‘Pearl Egg’. Moreover, this trend of increased starch and the total soluble sugar contents proved again that there a highly positive relationship exists between the side lighting and photosynthesis.

In our study, the lighting direction also affected the accumulation of soluble proteins in both cultivars (Figure 6C). For ‘Gaya Glory’, as the content of soluble proteins was the lowest with the bottom lighting, while the greatest value was observed with the side lighting. The soluble protein contents in ‘Pearl Egg’ displayed a similar tendency as in ‘Gaya Glory’.

### 2.7. Enzymatic Activity

**Figure 7:**
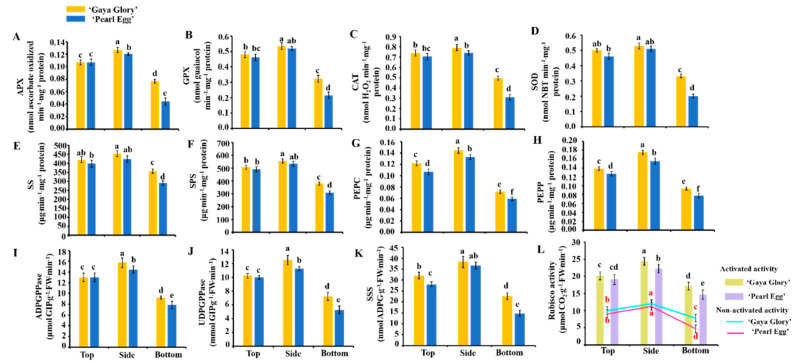
Changes in the enzymatic activities in chrysanthemum ‘Gaya Glory’ and ‘Pearl Egg’ as affected by the lighting direction after 45 days of cultivation. The ROS scavenging enzymatic activities: Ascorbate peroxidase (APX) (**A**), guaiacol peroxidase (GPX) (**B**), catalase (CAT) (**C**), and superoxide peroxidase (SOD) (**D**). Sucrose synthesis enzymatic activities: Sucrose synthase (SS) (**E**), sucrose synthase (SPS) (**F**), phosphoenolpyruvate carboxykinase (PEPC) (**G**), and phosphoenolpyruvate phosphatase (PEPP) (**H**). Starch synthesis enzymatic activities: Adenosine diphosphate glucose pyro-phosphorylase (ADPGPPase) (**I**), uridine diphosphate glucose pyro-phosphorylase (UDGPPase) (**J**), and soluble starch synthase (SSS) (**K**). Photosynthesis enzymatic activities: Activated and non-activated activity of RuBisCO (**L**). Vertical bars indicate the means ± standard error (*n* = 3). Different lowercase letters indicate the significant separation within treatments by Duncan’s multiple range test at *p* ≤ 0.05.

We further explore the effects of the lighting direction on the enzymatic activities in both ‘Gaya Glory’ and ‘Pearl Egg’ plants. The significant differences in the activity of ROS scavenging enzymes [ascorbate peroxidase (APX) (A), guaiacol peroxidase (GPX) (B), catalase (CAT) (C), and superoxide peroxidase (SOD) (D)], sucrose synthesis enzymes [sucrose synthase (SS) (E), sucrose synthase (SPS) (F), phosphoenolpyruvate carboxykinase (PEPC) (G), and phosphoenolpyruvate phosphatase (PEPP) (H)], starch synthesis enzymes [adenosine diphosphate glucose pyro-phosphorylase (ADPGPPase) (I), uridine diphosphate glucose pyro-phosphorylase (UDGPPase) (J), soluble starch synthase (SSS) (K)], and photosynthesis enzymes [activated and non-activated activity of RuBisCO (L)] were investigated under different lighting directions (Figure 7). As expected, all the aforementioned enzymatic activities decreased with the bottom lighting compared to those subjects to the top and side lighting, and the lowest values were measured with the bottom lighting in both ‘Gaya Glory’ and ‘Pearl Egg’. An acceleration in the activity of these enzymes occurred for both the top and side lighting, while the amplitude of acceleration was higher with the side lighting than with the top lighting. Taken together, these results suggest that the different enzymatic activities were directly associated with changes in the lighting direction. In our situation, using side lighting can be more effective at stimulating the enzymatic activities, regardless of the chrysanthemum cultivar.

### 2.8. Gene Expression

**Figure 8:**
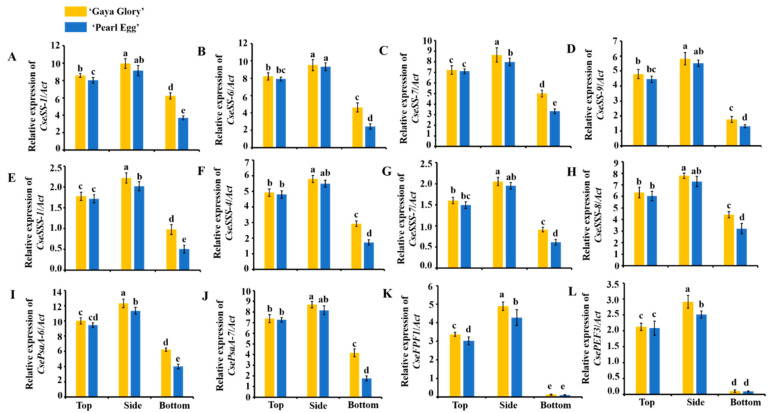
Changes in the gene expression levels in chrysanthemum ‘Gaya Glory’ and ‘Pearl Egg’ as affected by the lighting direction after 45 days of cultivation. Sucrose synthesis related genes: *CseSS-1* (**A**), *CseSS-6* (**B**), *CseSS-7* (**C**), and *CseSS-9* (**D**). Starch synthesis related genes: *CseSSS-1* (**E**), *CseSSS-4* (**F**), *CseSSS-7* (**G**), and *CseSSS-8* (**H**). Photosynthesis related genes: *CsePsaA-6* (**I**) and *CsePsaA-7* (**J**). Flowering related genes: *CseFPF1* (**K**) and *CsePEF3* (**L**). Vertical bars indicate the means ± standard error (*n* = 3). Different lowercase letters indicate significant separations within treatments by Duncan’s multiple range test at *p* ≤ 0.05.

Four of the genes involved in sucrose synthesis (*CseSS-1*, *CseSS-6*, *CseSS-7*, and *CseSS-9*), four genes involved in starch synthesis (*CseSSS-1*, *CseSSS-4*, *CseSSS-7*, and *CseSSS-8*), two genes involved in photosynthesis (*CsePsaA-6* and *CsePsaA-7*), and two genes involved in flowering (*CseFPF1* and *CsePEF3*) were selected from Plant GARDEN, available online: ‘https://plantgarden.jp/en/list/t1111766’ (accessed on 5 October 2021) and Mum GARDEN, available online: ‘http://mum-garden.kazusa.or.jp/’ (accessed on 5 October 2021), and the gene expression levels were determined in our study. The relative expression levels of all 12 genes in sucrose synthesis, starch synthesis, photosynthesis, and flowering were up-regulated with the light from the top and side as opposed to the bottom lighting (Figure 8). Strangely, the two genes related to flowering were barely expressed with the bottom lighting in both ‘Gaya Glory’ and ‘Pearl Egg’.

## 3. Discussion

### 3.1. Variations in Lighting Direction: Their Effects on Morphology and Growth Parameters of Whole Plant, Epidermal Cells, and Stomata

In this research, the two chrysanthemum cultivars of interest exhibited a consistent trend that varied to a degree, in response to the different lighting directions. The side lighting remarkably enhanced the shoot fresh and dry weights, and leaf area, but decreased the shoot length (Table 1), which is consistent with the results of a study that reported that in vitro micropropagated potato plantlets grown with a sideward lighting had significantly shortened stems but an increased dry weight and leaf area compared to those grown with the top (downward) lighting [53]. In addition, the greatest stem diameter, root length, and root fresh and dry weights of chrysanthemum observed with the side lighting may be regulated by higher photosynthesis, which supplies adequate energy to the stems and roots [54], and involves complex molecular regulation networks and endogenous plant hormones [55,56].

Interestingly, the lighting direction did not only affect the leaf morphology, root development, and plant biomass, but also had a significant influence on the induction of branches, stem nodes, and flowers in this study. Compared to the top and bottom lighting, the side lighting significantly increased the number of branches and stem nodes but reduced the internode length (Table 1). Apical dominance occurs when the terminal bud of the plant stem grows preferentially, and the auxin produced is polar-transported to the lateral bud while inhibiting the lateral bud production [57]. However, the opposite phenomenon was observed in plants grown with side lighting. We attempt to explain this phenomenon through two main theories. (1) The indirect inhibition theory of auxin [58,59,60]. The inhibition of lateral buds and shoots by auxin, whether naturally occurring or applied, is caused by a secondary inhibiting effect that arises from a primary auxin-promoted process in the main stem and travels upwards into the lateral buds and shoots, locations that auxin cannot easily access, such as cytokinin. In addition, it was found that the lateral buds were able to grow when the cytokinin was applied to the inhibited lateral buds, and the apical dominance was relieved. Therefore, an inhibition of the lateral buds occurred, because they did not have enough cytokinins directed toward them [61,62,63]. In our study, the chrysanthemum plants grown using the side lighting lacked light from the top and experienced a stunted development of the terminal bud. Due to the dysplasia of the terminal bud, the growth of the underside lateral buds was not inhibited by the production of sufficient auxins. Meanwhile, the well-developed lateral shoot preferentially receives cytokinins transported from the root, thus promoting the quantity and quality of the lateral bud. (2) In his nutrition theory, K. Gerber proposed the phenomenon of plant correlation inhibition in 1900, and concluded that the terminal bud cells grow rapidly, metabolize vigorously, and require more nutrients. Since the terminal bud preferentially feeds on nutrients transported by the roots and leaves, the lateral bud does not receive sufficient nutrients, thus experiencing stunted growth. In our experiment, the side lighting resulted in an inhibited growth of the main stem terminal bud due to insufficient light from the top. At the same time, the lateral bud received sufficient light, metabolized vigorously, which rapidly grew the cells, and in turn preferentially received more sufficient nutrients than the terminal bud, and their growth was further facilitated. Compared to the top and bottom lighting, the side lighting provided more favorable conditions for lateral bud induction and significantly enhanced the number of branches, nodes, and leaves. However, the relationship between hormone regulation and substance transport is complex and involves molecular-level interactions.

It is more remarkable that the side lighting induced abundant flowering in chrysanthemum plants (Figure 1A,B, and Table 1). Six flowering regulatory pathways have been identified in plants, including the photoperiodic pathway, vernalization pathway, temperature pathway, autonomous pathway, gibberellin pathway, and the age pathway. Phytochromes, cryptochromes, and ZTL/FKF1/LKP2 perceive light signals in leaves and transmit the signals to the circadian clock. After signaling integration via multiple flowering pathways, the photoreceptors eventually directly or indirectly regulate flowering [64,65,66,67]. Multiple photoreceptors are located on the upper leaf surface, which respond to the light of different wavelengths, and the resulting regulators are then transported from the phloem to the apical meristem, where they form a transcriptionally active flowering complex with a series of proteins that induces flowering [68]. The chrysanthemum plants grown with the side lighting possessed the greatest leaf area and number, capturing, and more efficiently using, the available light. This may explain why flowering was more significantly promoted by the side lighting.

Additionally, the greatest plant height, internode length, and the ratio of the shoot weight to the root weight (both fresh and dry weights) were observed for the top lighting (Table 1). Well-developed terminal buds inhibited the induction of lateral buds through apical dominance and were afforded easier access to nutrients. This, in turn, can lead to better health and nutrition, which can help to increase the plant height. In addition, plants will grow toward the top lighting due to phototropism. A significant increase of the shoot to root ratio was observed with the top lighting, compared to that with the side and bottom lighting, which could be explained by the negative phototropism of plant roots first discovered by Darwin [69]. Overall, these results demonstrated that the side lighting greatly improved the chrysanthemum morphology, and that side lighting is more important in increasing plant biomass, stem diameter, and for promoting branching and flowering compared to the top and bottom lighting.

Moreover, with the light from the bottom and the side, due to phototropism, the shoots generally display positive phototropism [26], whereby they bend toward the light so as to capture and more efficiently use the available light [27] (Figure 1A,B). The upper epidermal cells in the midribs were stimulated, and became prolate, while the wide flat cells were observed in the lower epidermis because of the squeezing (Figure 3), resulting from the increased adaxial leaf petiole angle and bent the leaves toward the light source (Figure 1C,D). The plants that were grown using the bottom lighting presented the largest leaf angle, followed by the plants grown with the side lighting (Figure 1J) and the bottom lighting also led to the lowest shoot height (Figure 1A,B).

Due to phototropism, leaf movement leads to changes in the leaf angle, and further stimulates the epidermal cells and stomatal state. In our study, the chrysanthemum plants grown with the side lighting exhibited the greatest density of small stomata, followed by the top lighting, while the plants grown with the bottom lighting had significantly less bigger stomata. Notably, the opened stomata were observed with the top and side lighting, more so with the side lighting, whereas the bottom lighting resulted in a closed pore (Figure 4). Consequently, lower photosynthetic efficiency and stress resistance may be observed in chrysanthemum plants grown with the bottom lighting due to the poor stomatal condition [32,33,34]. Thereby, the lowest levels of biomass production, plant growth, and development were observed with the bottom lighting (Table 1).

### 3.2. Variations in Lighting Direction: Their Effects on Leaf Anatomy, Chloroplast Distribution, and Chlorophyll Content

It is well known that the morphological structure and physiological functions of plants are unified, and the difference in the photosynthetic rate is closely related to the anatomical characteristics of photosynthetic organs in leaves. The results of this study have shown that an improved leaf structure can be obtained in chrysanthemum plants when grown with the top and side lighting, as compared to when they are grown with the bottom lighting. In addition, the side lighting mostly increases the leaf thickness and spongy tissue thickness, while the greatest palisade tissue thickness and the ratio of the palisade to spongy tissue thicknesses were observed for the top lighting. The greater leaf area ensured a high light interception capacity and photosynthetic efficiency. The photosynthetic rate was found to be sensitive to the leaf and the amount of carbon partitioned led to leaves growing thicker, which further promoted the development of the leaf structures [70,71]. The observed improvements in the thicknesses of leaves and spongy tissues with the side lighting may be linked with the well-developed mesophyll tissue [72]. The bottom lighting produced leaves with smaller cell sizes and loose cell layers, meaning that the thicknesses of the palisade tissues and spongy tissues were low, which may be due to the reduced cell growth and cell layer number in the mesophyll tissues [73]. Overall, the top lighting increased the palisade tissue elongation process, which enhanced the attachment region of the chloroplast, and the side lighting positively promoted the development of the spongy tissues that also contain chloroplasts and facilitates the passage of gases through its intercellular spaces [74]. Consequently, the thickness of the leaves and the photosynthetic capacity of the chrysanthemum leaves were significantly strengthened [75,76] with the side and top lighting.

One of the leaf traits most affected by photosynthesis is the chlorophyll content. In our study, significant changes were observed in the Chl a, Chl b, Chl a + b, and the ratio of the Chl a to Chl b contents, and the chlorophyll contents increased with the side lighting. These results were directly associated with the leaf thickness and chloroplast redundancy (Figure 2C and Figure 5). Our results are consistent with those reported in other studies [77,78].

On average, the differences in the leaf anatomy, chloroplast redundancy, and chlorophyll contents with the different lighting directions suggest that the structural components of leaves are the main targets of light, and by making adjustments in the leaf anatomy, plants are able to perform better with the side and top lighting.

### 3.3. Variations in Lighting Direction: Their Effects on Photosynthesis and Primary Metabolite Yields

In addition to the effects of the lighting direction on the morphology, leaf anatomy, and chloroplast arrangement, our findings demonstrate that the deleterious impacts of inappropriate lighting directions are diminished with optimum light conditions. In this study, the side and top lighting led to an enhanced net photosynthetic rate, stomatal conductance, intercellular carbon dioxide levels, and transpiration rate of chrysanthemum plants in comparison with the bottom lighting. Thus, this showed that the improved photosynthetic parameters enhanced the carbon gain and promoted chrysanthemum growth [79]. Moreover, these results suggest that an increase in the net photosynthetic rate which results from the side and top lighting may be due to the increase in the stomatal opening, and the changes in the net photosynthetic rate were closely associated with the stomatal opening [80,81,82].

An increased photosynthetic capacity is always accompanied by a high quantity of electrons passing through PSII [51]. Chlorophyll fluorescence characteristics are the main factors of photosynthetic regulation and plant responses to environmental conditions because of their sensitivity and convenience [83]. Chlorophyll fluorescence parameters are closely related to various reactions of photosynthesis and the effects of any stress on a certain process of photosynthesis can be reflected by the fluorescence kinetics of chlorophyllin [84]. Previous studies have reported that fluorescence parameters demonstrated a significantly positive linear relationship with the chlorophyll content in the living leaves of plants [85]. In the present study, similar results were obtained, and improved chlorophyll fluorescence characteristics were observed with the side and top lighting. These results reveal that optimum lighting directions enhance the efficiency of PSII and that they could enhance photosynthesis by improving the energy transport from PSII to PSI.

Furthermore, the lighting direction influenced the accumulation of the primary metabolites in both chrysanthemum cultivars. Carbohydrates, including starch and soluble sugars, are the direct expression of strong photosynthesis, and carbohydrate accumulation plays an important role in plant growth, development, and morphology [86]. The content of soluble proteins is an important physiological and biochemical index, and their content is an important indicator for understanding the overall metabolism of plants. Our data showed that the side and top lighting enhanced the carbohydrate and soluble protein levels (Figure 6), as a combined effect of the stomatal properties, chlorophyll contents, and use efficiency of light provided in different directions. These results were in general agreement with those of previous studies although small differences were observed, which may be due to the difference in the species [35]. The lighting direction played an important role in regulating the soluble protein levels and enzymes related to carbohydrates.

### 3.4. Variations in Lighting Direction: Their Effects on Enzymatic Activities and Gene Expression

The enzymatic activities of the key enzymes related to carbohydrate synthesis (Sucrose synthesis: SS, SPS, and PEPC; starch synthesis: ADPGPPase, UDPGPPase, and SSS) and photosynthesis (RuBisCO) significantly increased with the top lighting and even more so with the side lighting, as the highest activities of these enzymes were observed with the side lighting. In addition, changes in the lighting direction also played major roles in accelerating the activities of SS, SPS, and PEPC. Therefore, the plant biomass and net photosynthetic rate, which were largely regulated with the side lighting, may be affected by the activities of SS, SPS, PEPC, ADPGPPase, UDPGPPase, and SSS, and controlled the cell elongation and division in plants by regulating the expression of many genes (Figure 7E–K). These results indicate that the higher carbohydrate contents resulted from the association of these enzymes with other plant responses to the top and side lighting. In this case, plants grown with side lighting can be considered to be more effective at performing enzymatic activities. Furthermore, in this research, the activity of RuBisCO was increased with the side lighting (Figure 7L). The higher RuBisCO activity, which was promoted by the side lighting, showed that the higher net photosynthetic rate in chrysanthemum plants is directly correlated with the RuBisCO activity under changing environments [87]. In addition, to overcome the unfavorable conditions, plants have developed sophisticated acclimatization strategies, such as the ROS (reactive oxygen species) scavenged enzymatic antioxidant system [36]. ROS production is a common phenomenon in plants under stress. Under such circumstances, the balance between the ROS production and the quenching activities of antioxidants are disturbed, often resulting in oxidative damage [37]. Usually, a greater ability to eliminate ROS indicates higher antioxidant enzyme activities. Moreover, Chl a is more sensitive to ROS than Chl b, and under stress conditions, ROS directly caused the degradations of Chl a and the total chlorophyll contents [38,39]. In our experiment, the highly active ROS scavenging system involving peroxidase (POD), catalase (CAT), etc., occurred with the top and side lighting but not with the bottom lighting. The greatest enzyme activities were observed with the side lighting (Figure 7A–D). By comprehensively analyzing the previous results, the chlorophyll content was positively correlated with the activity of the ROS scavenging antioxidant system, and the side lighting effectively improved the chlorophyll content, the antioxidant capacity of the antioxidant enzyme system, and the resistance to stresses in chrysanthemums.

In our experiment, the side lighting led to the greatest up-regulation in gene expression. The relative expression levels of the four genes involved in the sucrose synthesis (*CseSS-1*, *CseSS-6*, *CseSS-7*, and *CseSS-9*) and the four genes involved in the starch synthesis (*CseSSS-1*, *CseSSS-4*, *CseSSS-7*, and *CseSSS-8*) were enhanced, and increased the production of total soluble sugars and starch to improve chrysanthemum growth and development (Figure 8A–H). The activities of sucrose synthesis enzymes (SS, SPS, PEPC, and PEPP), starch synthesis enzymes (ADPGPPase, UDGPPase, and SSS), and photosynthesis enzymes (RuBisCO) were directly related to the upregulation of these important genes. Furthermore, two genes involved in photosynthesis (*CsePsaA-6* and *CsePsaA-7*) were upregulated, and as the up-regulator of RuBisCO activation, the photosynthesis-related enzyme (Figure 8I,J) was also upregulated. It is remarkable that the two genes related to flowering (*CseFPF1* and *CsePEF3*) presented an extremely high performance, similar to flower induction in the chrysanthemum plants grown with the side lighting (Figure 8K,L). Therefore, in chrysanthemums, the specified genes acted as important regulators of carbon production, photosynthesis, and flower induction, and the side lighting led to improved growth.

## 4. Materials and Methods

### 4.1. Plant Growth and Treatment Design

The block-rooted cuttings of chrysanthemum (*Chrysanthemum morifolium* Ramat.) ‘Gaya Glory’ and ‘Pearl Egg’, a qualitative SD plant) were obtained from the Flowers Breeding Research Institute, Gyeongnam Agricultural Research & Extension Services (GARES), Republic of Korea, at the end of June 2021. The cuttings with 8 ± 1 leaves per plant were placed in 10 cm plastic pots containing a commercial medium (BVB Medium, Bas Van Buuren Substrates, EN-12580, De Lier, The Netherlands) and were acclimated for seven days on a glasshouse bench.

After acclimation, the transplanted seedlings were randomly divided into nine groups (each group contained 12 plants (six plants per cultivar)) and transferred into three separate plant growth chambers (C1200H3, FC Poibe Co., Ltd., Seoul, Korea), which acted as three repetitions, with a 25 °C temperature and 80% relative humidity. Each chamber was divided equally into three light-direction (top, side, and bottom) compartments using plates. The compartments with different lighting directions had a random distribution of positions in each chamber to avoid the occurrence of anu effects related to the position. In addition, all light-reflecting parts inside the chambers and the plates of each layer were wrapped with an opaque black curtain to prevent the different light directions from interacting. Each plate contained one group of plants, with 15 cm between each plant. The LED lamps used were custom made (SungKwang LED Co., Ltd., Incheon, Korea) and produced a wide spectrum, ranging from 400 to 720 nm with a distinct peak at 435 nm (blue) at a set light intensity of 300 μmol∙m^−2^∙s^−1^ photosynthetic photon flux density (PPFD) from 08:00 to 18:00 (SD condition) via an adjustment of the dimmer. Moreover, two modular-type LED lamps were placed 10 cm away from the top, side, or the bottom of the plants. Furthermore, the light intensity was measured using a quantum radiation probe (FLA 623 PS, ALMEMO, Holzkirchen, Germany) at the top-leaf level of the plant [35]. The plants were watered every day at 09:00 a.m. from 7 July to 15 August 2021 with a nutrient solution with a composition of (in mg∙L^−1^) 708.0 Ca(NO_3_)_2_∙4H_2_O, 246.0 MgSO_4_∙7H_2_O, 505.0 KNO_3_, 230.0 NH_4_H_2_PO_4_, 1.24 H_3_BO_3_, 0.12 CuSO_4_∙5H_2_O, 4.00 Fe-ethylene diamine tetraacetic acid, 2.20 MnSO_4_∙4H_2_O, 0.08 H_2_MoO_4_, and 1.15 ZnSO_4_∙7H_2_O [35]. Our study was not only designed as a completely randomized layout but was also composed of six biological replications with consistent growth to minimize the external influences.

### 4.2. Measurements of the Growth Parameters

After 40 days of cultivation, the growth parameters were measured. The plants were harvested and placed in liquid nitrogen in a −80 °C refrigerator for physiological investigations. Whole plants were obtained, and the roots were properly cleaned with tap water and severed from the shoot to measure the growth parameters. The shoot height, crown breadth, stem diameter, numbers of nodes and branches, the length of top 3rd internode, fresh weight, leaf number, length, width and angle, flower number, root length, and root fresh weight were measured directly. After drying for five days at 65 °C in a dry oven, the dry weights of the shoots and roots were measured. In addition, both the fresh and dry weights were used to calculate the shoot to root ratio.

### 4.3. Leaf Anatomical Features and Chloroplast Distribution

For each treatment, 10 leaf segments (1 cm^2^), without midribs, were collected from fully expanded leaves in the same stage for each treated plant. The segments were fixed in a formaldehyde solution containing 5% (*v*/*v*) formalin, 5% (*v*/*v*) acetic acid, and 90% (*v*/*v*) ethanol at 4 °C for three days. The leaf samples were dehydrated in a graded series of ethanol solutions (95, 75, 50, 25, and 10% (*v*/*v*) ethanol for each treatment for 40 min and for three repeats, respectively) and cut using the freehand slice method to an appropriate thickness. The sections were placed on glass slides and then directly observed with an optical microscope (ECLIPSE Ci-L, Nikon Corporation, Tokyo, Japan) without staining. The thicknesses of the total leaf, spongy tissues, and palisade mesophylls were measured with ImageJ.

The preestablished method was used to characterize the chloroplast distribution. Leaf pieces of the same size and replications were cut and fixed in a glutaric dialdehyde solution (4% (*v*/*v*) glutaraldehyde; 1% (*w*/*v*) osmium tetroxide) dehydrated in a graded series of acetone solutions and embedded in Epon812 [88]. Then ultrathin section was cut after uranyl acetate and lead citrate staining. A transmission electron microscope (H-600IV, TEM, Hitachi, Nagoya, Japan) was used for photographic examinations and measurements with ImageJ.

### 4.4. Epidermal Cell and Stomatal Characteristics

The upper and lower epidermal cells of leaves without midribs, as well as the abaxial surfaces of leaves, were carefully removed from the fully expanded leaves of three randomly selected plants in a similar position for each lighting direction to observe the epidermal cells. An observation of the stomata was obtained using transparent gummed tape to tear the epidermis from the leaf [89]. The excised samples were observed with an optical microscope (ECLIPSE Ci-L, Nikon Corporation, Tokyo, Japan) and analyzed with ImageJ. The number of stomata was divided by the area in which the number of stomata was recorded to obtain the stomatal density. The guard cell length, width of the guard cell pairs, and stomatal pore length and width were measured according with the directions provided by Sack and Buckley [31].

### 4.5. Photosynthesis and Chlorophyll Content

A leaf porometer (SC-1, Decagon Device Inc., Pullman, Washington, USA) was used to measure the photosynthetic parameters of fully expanded leaves at the same stage and position. All parameters, including the net photosynthetic rate (*P*_n_), transpiration rate (*T*_r_), stomatal conductance (*G*_s_), and intercellular CO_2_ concentration (*C*_i_) were measured inside the plant growth chambers to maintain the same steady condition from 9:30 to 11:30 in the morning.

The 0.1 g samples of fresh leaves were collected from each treated plant for chlorophyll content measurement for each treatment in six replicates. All of the samples were dipped in 10 mL of a N,N-dimethyl formamide solution in the dark for 48 h at 4 °C, and then both Chl a and Chl b were measured. The absorbances of the upper layer solution at 645 and 663 nm were recorded with a UV spectrophotometer (Libra S22, Biochrom Ltd., Cambridge, UK) as described in a previous study [90,91]. The chlorophyll content was calculated in accordance with the method described by Sim et al. [92]. The chlorophyll content was expressed as the chlorophyll/fresh leaf weights (mg/g).

### 4.6. Chlorophyll Fluorescence Measurements

The chlorophyll fluorescence measurement was performed with the miniaturized pulse-amplitude-modulated photosynthesis yield. Each plant was moved to a dark chamber for 30 min for adaption before being measured with a photo system (Fluor Pen FP 100, Photon Systems Instruments, PSI, Drásov, Czech Republic) to estimate the maximal PSII quantum yield (*F*v/*F*m), photochemical efficiency of PSII (*F*v′/*F*m′), non-photochemical quenching (NPQ), and the coefficient of photochemical quenching (*qP*). All the parameters were calculated using the methods reported by Maxwell et al. [93].

### 4.7. Contents of Carbohydrate and Soluble Protein

The samples of leaves were harvested at the same stage at the end of the day or night for carbohydrate measurements. The contents of starch and soluble sugars were determined by the Anthrone colorimetric method described by to Vasseur and Ren et al. [94,95]. The extraction method of soluble proteins followed our previous methodology whereby fresh leaves were collected, immediately immersed in liquid nitrogen, and were ground into a fine powder over an ice bath. A total amount of 100 mg of the powder was homogenized in 50 mM of PBS (1 mM EDTA, 1 mM polyvinylpyrrolidone, and 0.05% (*v*/*v*) triton-X, pH = 7.0). This mixture was then centrifuged (13,000 rpm, 4 °C, 20 min) to obtain the supernatant that would later be used for total protein estimation and the enzyme activity assay [96]. The total protein estimations were conducted using Bradford’s reagent [97,98]. The contents of both carbohydrate and soluble protein were measured with a UV spectrophotometer (Libra S22, Biochrom Ltd., Cambridge, UK).

### 4.8. Enzyme Activities

The antioxidant enzymes, including peroxidase (POD), catalase (CAT), superoxide dismutase (SOD), and ascorbate peroxidase (APX) were spectrophotometrically measured as described by Manivannan et al. [99].

The enzymatic activities of the key enzymes related to sucrose synthesis (SS, SPS, PEPC, and PEPP), starch synthesis (ADPGPPase, UDPGPPase, and SSS), and photosynthesis (RuBisCO) were measured using a UV spectrophotometer (Libra S22, Biochrom Ltd., Cambridge, UK). The SS and SPS were determined in a 1 mL reaction mixture containing a 500 μL enzyme extract at 34 °C for 1 h. A 300 μL 30% (*v*/*v*) KOH was added to this mixture and was then placed in a water bath at 100 °C for 10 min after which it was gradually cooled to room temperature. The mixture was subjected to incubation at 40 °C for 20 min after a 200 μL 0.15% (*v*/*v*) anthrone-sulfuric acid solution was applied and the enhancement of A_620 nm_ was monitored. The Rubisco total activity was measured by injecting 100 μL of the supernatant into 400 μL of an assay mixture consisting of 50 mM Tris-HCl (pH 8.0), 5 mM DTT, 10 mM MgCl_2_, 0.1 mM EDTA, and 20 mM NaH_14_CO_3_ (2.0 GBq mmol^−1^) at 30 °C. After a 5 min activation period, the reaction was initiated via the addition of RuBP to 0.5 mmol L^−1^ and was terminated after 30 s with 100 μL of 6 mol L^−1^ HCl. The PEPC was assayed in a 1 mL reaction mixture consisting of 50 mM Tris-HCl (pH 8.0), 5 mM MnCl_2_, 2 mM DTT, 10 mM NaHCO_3_, 0.2 mM NADH, 5 unit NAD-MDH, and a 160 μL enzyme extract. The reaction was initiated by adding 2.5 mM phosphoenolpyruvate (PEP). The PEPP was determined in a 1.5 mL reaction mixture containing 100 mM imidazole-HCl (pH 7.5), 50 mM KCl, 10 mM MgCl_2_, 0.05% (*w*/*v*) BSA, 2 mM DTT, 150 μM NADH, 1 unit LDH, 2 mM ADP, and a 150 μL enzyme extract. The reaction was initiated with 2 mM PEP, and the increase in the A_412 nm_ was monitored. The above description of enzymatic activities was conducted in accordance with the directions provided by Feng et al. and Yang et al. [100,101]. In addition, activities of adenosine diphosphate glucose pyro-phosphorylase (ADPGPPase), uridine diphosphate glucose pyrophosphorylase (UDPGPPase), and soluble starch synthase (SSS) were measured according to the protocol described by Doehlert et al. and Liang et al. [102,103].

### 4.9. Real-Time Quantitative PCR Verification

The expanding leaves of both ‘Gaya Glory’ and ‘Pearl Egg’, of chrysanthemum at the same stage were harvested on six plants with three lighting directions for a consideration of the RNA abundance and the sensitivity of the blade to lighting. The expression of key genes related to sucrose synthesis (*CseSSS-1*, *CseSSS-4*, *CseSSS-7*, and *CseSSS-8)*, starch synthesis (*CseSS-1*, *CseSS-6*, *CseSS-7*, and *CseSS-9*), photosynthesis (*CsePsaA-6* and *CsePsaA-7*), and flowering (*CseFPF1*, *CsePEF3*, *CsePEF4*, and *CseFCA*) were measured. All the leaves were immediately frozen in liquid nitrogen. The total RNA was extracted using an Easy-Spin total RNA extraction kit (iNtRON Biotechnology, Seoul, Korea), which was then used for a first-stand cDNA synthesis with the GoScript Reverse Transcription System (Promega, Madison, WI, USA) according to the manufacturer’s protocols. A real-time quantitative PCR was conducted in a real-time PCR system (CFX96, Bio-Rad, Hercules, CA, USA). T reaction volumes (20 μL) contained 1 μL of cDNA, 1 μL of each amplification primer (10 μM), 10 μL of 2 × AMPIGENE qPCR Green Mix Lo-ROX (Enzo Life Sciences Inc., Farmingdale, New York, NY, USA), and 7 μL ddH_2_O (double distilled water). The 2−ΔΔCt method was used for the data analysis, and the *ACTIN* gene (Cse_sc001321.1_g010.1) was selected as the control. All the target gene primers are listed in Table 4.

### 4.10. Statistical Analysis

The notable differences among the treatments were assessed via an analysis of variance (ANOVA) followed by Duncan’s multiple range test at a probability of (*p*) < 0.05 using a statistical program (SAS, Statistical Analysis System, V. 9.1, Cary, NC, USA). The Fisher’s least significant difference test was used for the F-test between treatments. The experimental assays that were used to obtain all of the results were repeated three times and are presented as the mean ± standard error.

## 5. Conclusions

The significant effects of lighting on plants have been extensively investigated, as studies have rarely reported the impacts of different lighting directions on chrysanthemum, to understand the optimum requirement of lighting direction for better growth and development. This study demonstrated that the leaf angle, which is adjusted by the lighting direction, strongly regulated the morphophysiology of the plant by adjusting the capture and efficiency of the available light in chrysanthemums. The top lighting, and to a greater extent, the side lighting significantly improved the morphological parameters, carbon assimilation rate (production of soluble sugar and starch) and the enzymatic activities of key enzymes by up-regulating the essential carbohydrate synthesis and photosynthesis related genes. Moreover, the higher levels of flower induction that occurred with the side and top lighting were up-regulated by flowering-related genes of chrysanthemum (*CseFPF1* and *CsePEF3*). In addition, compared to the bottom lighting, the top and side lighting improved the leaf structure and anatomy, which in turn significantly increased the photosynthesis and chlorophyll fluorescence, especially in the quantum yield of PSII, which in turn, substantially enhanced the chrysanthemum growth and development. Therefore, as observed in our results, the top lighting and to a greater extent, the side lighting provide the optimum lighting direction, which altered the leaf orientation and adjusted the leaf angle to grow toward the light to capture and more efficiently use the available light and induced branching and flowering. It can therefore be concluded that chrysanthemum plants grow well under prevailing conditions. Further investigation remains necessary to explore the differences in the internal hormone distributions and concertation between two sides of the curved sections of the stem or leaf. The regulation networks of plant growth and development are quite complex, involving not only environmental factors but also plant hormones, active proteins and genes, and even different responses to different cultivars or species.

## Figures and Tables

**Table 1:** Influence of the lighting direction on the growth and development of chrysanthemum grown for 45 days.

Cultivar (A)	Lighting Direction (B)	Shoot			Leaf			Flower		Root			Shoot/Root (FW)	Shoot/Root (DW)
		Length of Internode (mm)	Fresh Weight (g)	Dry Weight (g)	Number	Length (cm)	Width (cm)	DVB ^1^ (Day)	Number	Length (cm)	Fresh Weight (g)	Dry Weight (g)		
‘Gaya Glory’	Top	6.1 a ^2^	32.6 c	2.1 c	77.3 b	3.5 d	2.4 d	23.3 a	47.0 b	31.8 c	2.4 c	0.24 c	13.6 a	8.8 a
	Side	5.7 c	40.0 a	3.0 a	95.7 a	3.7 c	2.6 c	23.3 a	61.7 a	38.8 a	3.6 a	0.34 a	11.1 b	8.5 b
	Bottom	4.0 e	6.7 e	0.5 e	43.0 d	3.8 b	1.9 e	-	0.0 e	11.8 d	0.8 e	0.08 e	8.4 d	6.3 e
‘Pearl Egg’	Top	5.9 b	24.8 d	2.0 d	66.3 c	3.2 e	3.0 b	20.7 b	20.7 d	32.2 c	2.1 d	0.23 d	11.8 b	8.7 a
	Side	5.3 d	34.4 b	2.9 b	77.7 b	3.7 c	3.2 a	20.7 b	38.7 c	37.0 b	3.5 b	0.32 b	9.8 c	8.2 c
	Bottom	3.1 f	4.1 f	0.5 e	23.3 e	3.9 a	1.8 f	-	0.0 e	10.7 e	0.6 f	0.07 e	6.8 e	7.1 d
*F*-test	A	***	***	***	***	***	***	***	***	***	***	***	**	***
	B	***	***	***	***	***	***	NS	***	***	***	***	***	***
	A x B	***	***	**	*	***	***	NS	***	***	***	***	***	***

^1.^ Days of treatment to the visible flower buds. ^2.^ Mean separation within columns by Duncan’s multiple range test at *p* ≤ 0.05. NS, *, **, ***, non-significant or significant at *p* ≤ 0.05, 0.01, or 0.001, respectively.

**Table 2:** Influence of the lighting direction on the photosynthetic characteristics of chrysanthemum grown for 45 days.

Cultivar (A)	Lighting Direction (B)	*P*_n_ ^1^ (μmol CO_2_ m^−2^·s^−1^)	*T*_r_ ^2^ (mmol H_2_O m^−2^·s^−1^)	*G*_s_ ^3^ (mol H_2_O m^−2^·s^−1^)	*C*_i_ ^4^ (μmol CO_2_ mol^−1^)
‘Gaya Glory’	Top	15.72 b ^5^	1.77 b	0.72 a	467.41 b
	Side	16.61 a	1.86 a	0.74 a	479.47 a
	Bottom	12.25 d	1.27 d	0.35 c	393.67 c
‘Pearl Egg’	Top	14.22 c	1.53 c	0.66 b	463.80 b
	Side	14.51 c	1.87 a	0.66 b	467.64 b
	Bottom	11.40 e	1.11 e	0.32 c	350.42 d
*F*-test	A	***	**	***	***
	B	***	***	***	***
	A × B	**	NS	NS	NS

^1.^ Net photosynthetic rate. ^2.^ Transpiration rate. ^3.^ Stomatal conductance. ^4.^ Intercellular CO_2_ concentration. ^5.^ Mean separation within columns by Duncan’s multiple range test at *p* ≤ 0.05. NS, **, ***, non-significant or significant at *p* ≤ 0.01 or 0.001, respectively.

**Table 3:** Influence of the lighting direction on the chlorophyll fluorescence characteristics of chrysanthemum grown for 45 days.

Cultivar (A)	Lighting Direction (B)	*F*_v_/*F*_m_ ^1^	*F*_v_’/*F*_m_’ ^2^	NPQ ^3^	*qP* ^4^
‘Gaya Glory’	Top	0.97 a ^5^	0.71 a	2.84 a	0.58 a
	Side	0.99 a	0.72 a	2.87 a	0.59 a
	Bottom	0.80 d	0.52 c	2.37 c	0.44 c
‘Pearl Egg’	Top	0.91 b	0.67 b	2.78 b	0.53 b
	Side	0.92 b	0.68 b	2.82 ab	0.57 a
	Bottom	0.75 c	0.43 d	1.93 d	0.36 c
*F*-test	A	***	***	NS	**
	B	**	**	**	**
	A × B	***	***	**	NS

^1.^ The maximal PSII quantum yield (*F*_v_/*F*_m_). ^2.^ The photochemical efficiency of PSII (*F*_v_′/*F*_m_′). ^3.^ Non-photochemical quenching (NPQ). ^4.^ Coefficient of photochemical quenching (*qP*) ^5.^ Mean separation within columns by Duncan’s multiple range test at *p* ≤ 0.05. NS, **, ***, non-significant or significant at *p* ≤ 0.01 or 0.001, respectively.

**Table 4:** List of the primers used to quantify expression levels of genes.

Name	Gene ID	GO/Pfam ID	Primer Sequence (5’ to 3’)
*ACTIN* (Cse_sc001321.1_g010.1)	3641_0:00292c	GO:0005524	F: AACTGGGACGATATGGAGAAGA
			R: CGCAAGATAGCATGTGGAAGTG
*CseSS-1* (Cse_sc003876.1_g050.1)	4232_0:003231	GO:0009011	F: GACCCCGGTGGAAATAGTGA
			R: TTGCAAGGCCTCTTTCTCAGT
*CseSS-7* (Cse_sc012707.1_g010.1)	4232_0:0027ec	GO:0009507	F: GGCCTTGGAGCAAAACTGGT
			R: AGTCTATTCCAGCAACAGGTCC
*CseSS-9* (Cse_sc009929.1_g030.1)	4232_0:0076eb	GO:0004373	F: TCCGTACTTCAGACGCCAATC
			R: GTTTCGACCCAGTTCCCATC
*CseSS-6* (Cse_sc029166.1_g010.1)	4232_0:000861	GO:0009059	F: CCAAACCAAGCAGTCCAAGAA
			R: TACGCAACTCTTCTTCCATTTGT
*CseSSS-4* (Cse_sc005354.1_g010.1)	4232_0:003c28	GO:0016157	F: TGAGAATATGTGCTGGCGGA
			R: TCGCACCAACCCATGGATAC
*CseSSS-7* (Cse_sc001317.1_g020.1)	4232_0:004d5f	PF00534	F: ACGTTGCATTAGGGGTACGA
			R: GCAGCGGTTTTGCATTCTCT
*CseSSS-8* (Cse_sc008612.1_g030.1)	4232_0:002593	GO:0016157	F: TGCCCCCGTTTGTAGCTTTA
			R: TCCAGGAGTGGCTCCAAACA
*CseSSS-1* (Cse_sc001888.1_g140.1)	4232_0:009180	GO:0016157	F: TATTCGTCTTCGTCCCGGTG
			R: TGGTGAGTACGGAGGAAATCG
*CsePsaA-7* (Cse_sc003237.1_g010.1)	4232_0:00aa9f	GO:0016021	F: ACTGGTAGTGGTGGGAAAGC
			R: CTTAGAGCCTGAGCATCTGAGT
*CsePsaA-6* (Cse_sc022053.1_g010.1)	4236_0:0065f0	PF14870	F: GGAAAGCCAACCAAATGATGCT
			R: TCCTCGGTTATAAGCAGCCAC
*CseFPF1* (Cse_sc015873.1_g020.1)	4236_0:004cfa	GO:0009909	F: ATGTCTGGTGTTTGGGTGTTTA
			R: CTACATATCTTACTTCAA
*CsePEF3* (Cse_sc001254.1_g140.1)	4232_0:0032f4	GO:2000028	F: ATGTCGTTTAACGTACCATCACAA
			R: ATCACCACGTTTCAGCTGTCC
*CsePEF4* (Cse_sc001459.1_g030.1)	4232_0:00932a	GO:0042753	F: GATGGCAAGGTGATGCAAACA
			R: TCGAAAAATTCGACGAAAGATCC
*CseFCA* (Cse_sc021505.1_g020.1)	4232_0:0028ef	PF00076	F: GGTCATACGACAACTACGGC
			R:AGTTCTTGAAAAGAATAACCTCGG

## Data Availability

Data sharing is not applicable to this article.
